# Plasma fibrinogen degradation products with salivary coagulopathy in OSMF among Indians

**DOI:** 10.6026/973206300200829

**Published:** 2024-08-31

**Authors:** Kanika Sethi, Narendra Nath Singh, Ankita Tandon

**Affiliations:** 1 Department of Oral Pathology and Microbiology, Inderprastha Dental College, Sahibabad, Ghaziabad, Uttar Pradesh, India; 2 Department of Oral Pathology, Microbiology and Forensic Odontology, Dental Institute, RIMS, Ranchi, Jharkhand, India

**Keywords:** Oral submucous fibrosis, salivary coagulopathy, fibrinogen degradation products

## Abstract

Oral submucous fibrosis (OSMF) produces changes localized to oral cavity. Hence, it is reasonable to assume that saliva may have an important role in its etiology. This prospective case control study included 37 patients with OSMF which were compared with age and gender matched healthy controls. Salivary coagulopathy procedures as described by Chatuvedi *et al.* and quantification of plasma fibrinogen degradation products using a commercially available kit were carried out. The obtained data was analyzed using Chi-square tests and Tukey HSD test. The salivary coagulopathy was strongly indicated in saliva of OSMF patients and was dependent on the severity of disease. There were also increased levels of plasma fibrinogen degradation products compared to the controls and were found to have a statistically significant correlation with increasing clinical grades of oral submucous fibrosis (p≤ 0.05)

## Background:

Oral Submucous Fibrosis (OSMF) is a premalignant and crippling condition of the oral mucosa. The characteristic features of the disorder include submucosal fibrosis leading to secondary atrophic changes in the epithelium. The mucosa appears pale or whitish, mottled or opaque white and feels hard and board like. The atrophic epithelium becomes sensitive to spicy and hot food and also becomes vulnerable to carcinomatous changes [1]. This is undoubtedly a classic "Disease of Civilization" with large differences seen between races, geographic areas and individuals at different levels in both prevalence and the degree to which it transforms into malignancy [1]. The possible premalignant nature of the disease was first emphasized by Paymaster who described among with OSMF patients in Bombay, the development of slow growing squamous cell carcinoma [2]. The malignant transformation rate of OSMF is reported as 2.3% to 7.6% [3]. This condition affects approximately 0.5% (5 million people) of the population in the Indian subcontinent, and following migration from this region, is now a healthcare problem in many parts of the world [4]. Since the disease is mainly in the South East Asian countries, extensive studies have been undertaken at different centers in this part of the globe to define the etiology of the disease. Earlier studies were mainly done to establish the role of local irritants like betel quid (Pan chewing and/ or bidi smoking) or chilies used in cooking and secondly, to correlate various vitamin deficiencies and malnutrition with OSMF. This however did not explain the development of OSMF in those individuals who did not have any habits and who did not suffer from hypovitaminosis or malnutrition. At the same time, this did not explain the almost exclusive occurrence of OSMF in South East Asians [5]. It is strongly believed that saliva may have a strong role in causation of the disease as the changes are restricted to oral mucosa. A thrombin like, fibrin producing factor is often described in saliva of patients suffering from OSMF which when encountered the fibrinous exudates in the oral cavity promptly clotted it. Also, an increased production of fibrinogen with increased fibrin deposition has been reported in literature. The catabolic breakdown of this increased fibrinogen results in increased levels of fibrinogen degradation products which could be assessed in the plasma [5- 6]. Therefore, it is of interest to report interplay between systemic abnormality and local factors which might be responsible for this localized precancerous condition.

## Materials and Method:

The present case control study was conducted on 74 subjects visiting the OPD after informed consent from patients and ethical clearance from Institutional Review Board. Out of 74 subjects, 37 were clinically diagnosed as Oral sub mucous fibrosis and 37 were taken as controls. Patients with any systemic diseases such as hepatosplenomegaly, bleeding & clotting disorders were excluded from the study.

## Clinical Examination and Study parameters:

A detailed history was recorded regarding duration and frequency of habits such as areca nut, pan chewing with/ without tobacco, gutkha chewing etc. A careful observation of symptoms like burning sensation to normal or spicy food and its duration, difficulty in swallowing and speech and its duration, increased or decreased salivation with altered taste sensation and its duration was also made. The study subjects were grouped according to clinical staging as described by Ghom A (2007) [7].

## Saliva collection:

10 ml of expectorated saliva after thorough rinsing of oral cavity was collected in a graduated glass cylinder and was centrifuged at 1000 rpm for 10 minutes [8].

## Plasma collection:

Under all aseptic conditions, 4.5 ml of collected blood was mixed with 0.5 ml of anticoagulant EDTA solution (ratio 1:5). The sample was kept in refrigerator for 1 hour and then centrifuged for 10 minutes at 3000 rpm. The plasma thus obtained was used for coagulopathy procedure and FDP quantitation.

## FDP Qualitation and semi quantification procedure:

**Figure 2:**
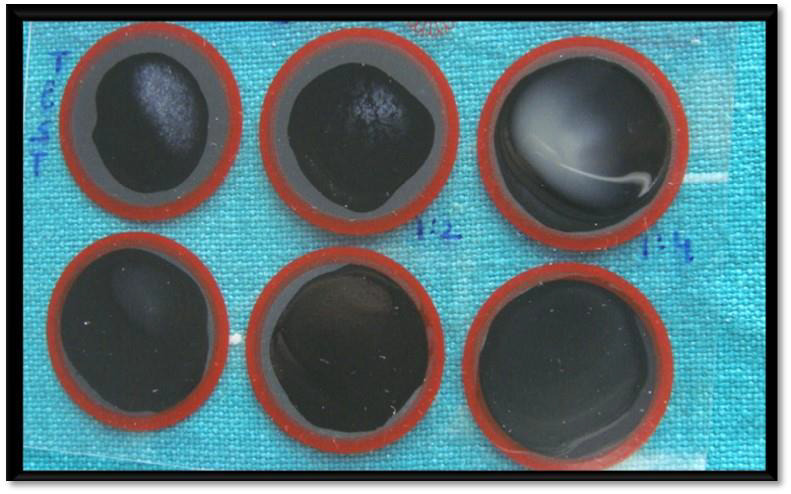
FDP quantification using a commercially available kit employing quantification.

Tulip XL FDP- A qualitative and semi-quantitative latex slide test for detecting cross linked fibrin degradation products in human plasma by Tulip Diagnostics P. Ltd., Goa, India was used for the procedure. XL FDP slide test for detection of cross-linked fibrin degradation products is based on the principle of agglutination. The test specimen (plasma) is mixed with XL FDP latex reagent. The sensitivity of the reagent is ~200ng/ml, below which samples are negative and above which samples give a positive agglutination reaction. The cross-linked fibrin degradation products, D dimer, D dimer E, and high molecular weight derivatives are all recognized by the XL FDP reagent incorporating the monoclonal antibodies. No binding is found to the fibrinogen degradation products X, Y, D and E to 20 mg/L or to fibrinogen up to 1000 mg/L. For the test, all the kit reagents and plasma samples were brought to room temperature before performing the test. One drop of the plasma specimen was pipetted onto the plastic slide. To this one drop of XL FDP latex reagent was added adjacent to the plasma specimen. The plasma specimen and the latex reagent were uniformly mixed over the entire circle using a mixing stick. The slide was gently rocked back and forth for three minutes and the agglutination was macroscopically observed. Using PBS buffer solution, provided with the kit, serial dilutions of the plasma samples were prepared in test tubes as 1:2, 1:4, 1:8, 1:16. and so on. One drop of latex reagent was mixed with a drop of diluted plasma specimen on different circles taking care to avoid cross contamination. The slide was gently rocked back and forth for three minutes and the agglutination was macroscopically observed. Presence of agglutination was a positive qualitative result indicating D dimer levels above 200ng/ml. To semi-quantitatively assess the levels of D dimers in the specimen, agglutinations in the highest plasma dilution samples were taken into consideration. To calculate the D dimer levels, the following formula provided by the manufacturer was used (Figure 2).

D dimer level (ng/ml) = 200 x d

d = highest dilution of plasma showing agglutination during the semi quantitative test of the sample.

## Results:

## Distribution of cases in study and control group:

Maximum number of subjects in Study Group were in Stage II (14.9%) followed by Stage III (13.5%). An equal number of subjects were in Stage I and Stage IV (n=8; 10.8%) respectively.

## Age and Gender wise distribution of subjects:

In both the groups, majority of subjects (54.1%) were upto 30 years of age and majority (83.8%) of them were males. Only 16.2% subjects in each group were females.

## Assessment of Salivary coagulopathy:

## Clinical staging and coagulopathy:

**Figure 3:**
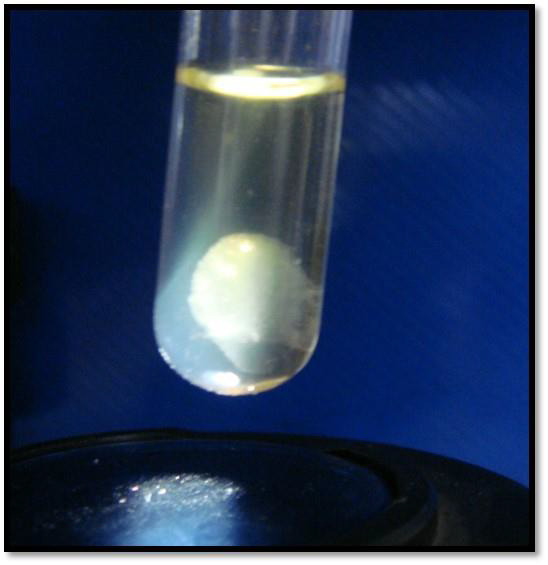
Salivary coagulum.

The formation of coagulum was observed in 50% (4) cases of Stage I, 63.6% (7) cases of Stage II, 100% (10) cases of Stage III and 100% (8) cases of Stage IV. Significantly higher proportion of subjects with higher stages had positive coagulopathy (p=0.017). In stage III and IV all the subjects had positive coagulopathy (Figure 3).

## Salivary coagulopathy and duration:

Since, the median value for duration of OSMF in study group was found to be 7 years hence the data was pooled into two groups to see the Coagulopathy. Though the proportion of subjects with positive coagulopathy was higher in >7 years duration group, yet no statistically significant association between duration of OSMF and coagulopathy could be established (p=0.131).

## Comparison of salivary coagulopathy in cases and control groups:

## Using Normal saliva:

At 37°C, within Control group no coagulopathy was seen. In Study group maximum coagulopathy (78.4%) was observed for t1 condition followed by t4 condition. For t2 and t3 conditions no coagulopathy was seen. Statistically, a significant difference between two groups was seen for t1 and t4 conditions.

At 4°C too, in Control group no coagulopathy was seen for any condition. In study group, maximum coagulation (55.6%) was seen for t1 test tube followed by t4 test tube condition (18.9%). No coagulopathy was seen for t2 and t3 test tubes. Statistical comparison revealed a significant difference between two groups for t1 and t4 conditions.

## Using Boiled Saliva:

No coagulopathy was seen for any of the specimen of boiled saliva specimen in control and study group for any condition at 37°C and 4°C.

## Comparison of salivary coagulopathy in different clinical stages of OSMF:

As a trend, incidence of coagulopathy was seen to be lower in lower stages and higher in higher stages. No coagulopathy was seen for t2 and t3 test tubes. Statistically, there was a significant difference in incidence of coagulopathy among different stages for t1 and t4 conditions (Table 1).

## Assessment of FDP levels:

## Comparison of FDP expression in study and control groups:

No FDP expression was seen in any of the control group while in study group, expression of FDP was observed in 35/37 (94.6%) cases (p<0.001).

## Association of FDP expression and clinical staging:

Except stage I, for all the stages all cases had positive FDP expression. In stage I the positivity was only 25%. However, inter stage difference was not found to be significant statistically (p=0.053).

## Association of FDP Quantification and clinical staging:

**Figure 4:**
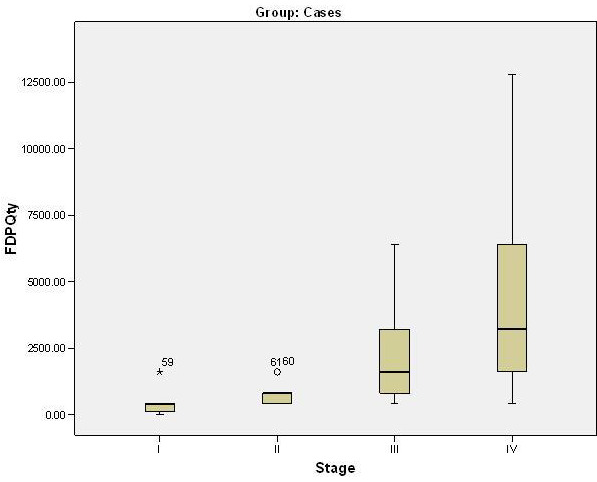
Association of FDP quantification and clinical staging.

Stage I and Stage II had mean FDP values of 425.0±506.4 and 763.6±454.5 which were significantly lower as compared to Stage III and Stage IV with mean values of 2360.0±2266.3 and 4450.0±4019.6 respectively (F=4.439; p<0.001) (Figure 4).

## Multiple comparisons of FDP levels in various clinical stages:

Multiple comparisons revealed no statistically significant difference between Stage I and Stage II and between Stage III and Stage IV. However, both Stage I and Stage II had significantly lower mean value as compared to Stage IV. A statistically significant difference was seen between Stage I and Stage III too. No other comparison was significant statistically (p>0.05).

## Diagnostic Efficacy of Salivary Coagulopathy and Fibrinogen Degradation Product Quantification:

## Between Control and study groups:

**Figure 5:**
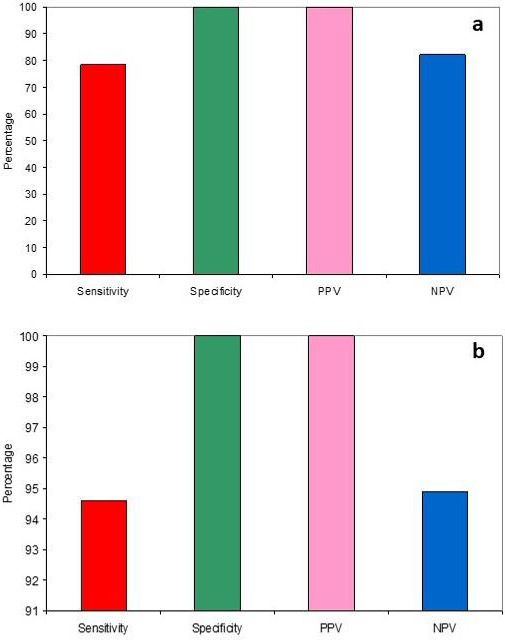
Diagnostic efficacy of salivary coagulopathy (a) and FDP estimation (b).

Since the incidence of coagulopathy was high in test tube t1 at 37°C in the study group, hence it was chosen as the reference marker. It was seen that coagulopathy was 78.4% sensitive and 100% specific with a positive predictive value of 100% and a negative predictive value of 82.2% (Figure 5a). FDP expression was found to be 94.6% sensitive and 100% specific with a positive predictive value of 100% and a negative predictive value of 94.9% (Figure 5b).

## Between various clinical stages in study group:

**Figure 6:**
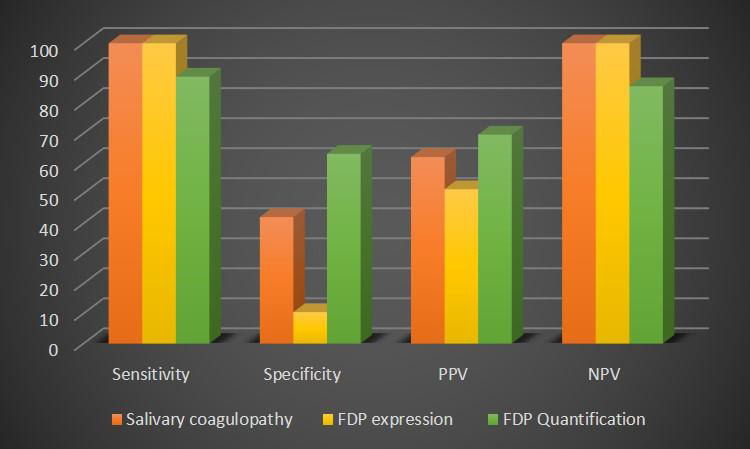
Correlation between diagnostic efficacy of salivary coagulopathy, FDP expression and FDP quantification among various clinical stages in study group.

The sensitivity was found to be 100% while the specificity was only 42.1%. The positive predictive value was 62.1% and negative predictive value was 100%. FDP expression was found to be 100% sensitive and 10.5% specific with a positive predictive value of 51.4% and a negative predictive value of 100% (Figure 6).

## Diagnostic efficacy of FDP quantification among various clinical stages:

For study group, the median FDP value was 800, hence it was taken as the cut-off point. FDP level >800 was found to be 88.9% sensitive and 63.2% specific with a positive predictive value of 69.6% and negative predictive value of 85.7% (Figure 6).

## Correlation of FDP expression in relation to salivary coagulopathy:

A high level of agreement (k=0.836) was seen between salivary coagulopathy and FDP expression. FDP expression was found to be 100% sensitive and 86.7% specific to the coagulopathy findings and had an 82.9% PPV and 100% NPV for coagulopathy findings (Table 2).

## Discussion:

Oral submucous fibrosis (OSMF) is an enigmatic disease of unknown etiology, reported mainly in Indians hallmarked by the presence of submucosal fibrosis which affects most parts of the oral cavity, pharynx and upper third of esophagus. The etiology of OSMF is varied. In spite of intense work no single factor has been pointed out as being the only causative factor in OSMF [10]. A number of factors trigger the disease process by causing a juxta-epithelial inflammatory reaction in the oral mucosa. Factors include areca nut chewing, ingestion of chilies, genetic and immunologic process, nutritional deficiencies and other factors. The chewing of betel quid (containing areca nut, tobacco, slaked lime or other spices has been recognized as one of the most important risk factors for OSMF as supported by epidemiological evidence [11]. In the present study, OSMF subjects were in the age range of 18-80 years with a mean age of 33.5 years. Maximum cases i.e 28(75.6%) were in the age range of 20-40 years. Similar affected age range of OSMF patients were also quoted in other studies [1, 10, 12, 13, 14, 15, 16, 17, 18, 19, 20- 21]. Present study also indicated that most of the younger people were suffering from OSMF. This could be attributed to the advent of attractive, conveniently packed sachets and mass and media advertisements, consuming of gutkha and pan masala by younger people. The other reason might be an easier and sophisticated alternative of indulging into addictive habit in working arenas where smoking is banned in public areas by the government lately without putting any restrictions to spitting. In our study, of the 37 OSMF patients, 31 (83.8%) were males and 6 (16.2%) were females with male to female ratio of 5:1. A male predominance has also been reported other authors [1, 22, 23-24]. This is contrary to the findings of studies [5, 25, 26- 27] where a female predilection has been reported. This gender variance could probably be due to the fact that the present study was conducted in dental settings where the sample is constrained to a selective referral centre only with the habit more commonly seen amongst males and males seeking more dental care at tertiary level. In the present study, formation of coagulum was seen in 78.4% cases. This was in agreement with [5] and [8] who observed salivary coagulopathy in 69.9% and 78.33% cases of OSMF respectively. The coagulum was white in colour and was suspended in the upper part of the test tube. The coagulum was present in test tube 1(where patient's saliva and patient's plasma samples were diluted with normal saline) and test tube 4 (where patient's saliva was mixed with control plasma and diluted with normal saline) only but the size was variable in both the test tubes and at 37°C and 4°C.

Chatuvedi *et al.* [5] in their study reported a similar observation in the saliva of OSMF patients. While the exact origin of this precipitate is not known, the presence of elevated levels of plasma fibrinogen and fibrin precipitating factor in saliva of OSMF patients could be considered as a causative agent in the formation of salivary coagulum. It was thus hypothesized that an increased consumption of fibrinogen in the oral cavity was responsible for its elevated levels in the plasma and may represent transformation products in the conversion of fibrinogen to fibrin. The presence of fibrin precipitating factor in saliva along with altered fibrinogen metabolism could be considered to explain the localization of this fibrotic process only to the oral cavity. However, when the boiled saliva was used to test the coagulopathy procedure, no coagulum formation could be appreciated. These findings were similar to [5] and [8]. It is thus indicated that the coagulum is heat labile and protein in nature. There was also a progressive increase in percentage of cases with increase in the severity of the disease showing coagulum formation in saliva and this was found to be statistically significant (p= 0.017). In the present study there was progressive increase in percentage of cases with increase in severity of disease showing coagulum formation in saliva which was similar to the findings of Pinakapani *et al.* (2009) [8]. Also, the increase in the quality of coagulum with ascending grades of the disease could be attributed to the increased consumption of fibrinogen in the oral cavity and hence increased production of fibrinogen in liver thus resulting in elevated levels of plasma fibrinogen.

The plasma FDP levels of 37 OSMF patients and 37 control subjects were quantitated using a commercially available kit in which the sensitivity of the reagent is ~200ng/ml, below which samples are considered negative. Amongst controls, the plasma FDP levels were below the detectable levels and hence the levels could not be assessed. Our findings were similar to the observations of Pathak AG (1984) [28], Koshti S and Barpande S (2007). In our study, except for stage I, all the stages had a positive FDP expression. In grade I, the positivity was 25% only. The negative findings of our study in stage I could be due to low levels of FDP in these patients, probably below detectable levels in the early stages of the disease mainly manifesting stomatitis. The mean plasma FDP levels in various clinical stages of OSMF was 425.0 ng/ml in Stage I, 763.6 ng/ml in Stage II, 2360.0 ng/ml in Stage III and 4450.0 ng/ml in Stage IV. A significant inter-stage difference was also seen for FDP levels which progressively increased with the increasing stage of the disease (p< 0.001). The abnormal levels of FDP, above the normal estimable levels are a sign of hypercoagulable state of the disease thus resulting in increased fibrinolysis. The altered FDP levels could be an early diagnostic sign of increased fibrin deposition with an altered repair mechanism taking place thus resulting in increased collagen deposition. This increased abnormal collagen deposition could further accentuate the disease process resulting in progression of the fibrosis and thus the severity of the disease [9].

The literature states that FDP is an early diagnostic sign of fibrin deposition, the increase in its level suggests that there is increased fibrin deposition in OSMF. Thus, the findings that OSMF is primarily a change of connective tissue are further strengthened. Moreover, with an increase in clinical grade, plasma FDP levels are also increased. Thus, suggesting that as the clinical grade increased, the amount of fibrin deposited in the connective tissue also increased, leading to progressive restriction in mouth opening. Our study results showed that salivary coagulopathy was a good marker for differentiation between control and OSMF patients with 78.4% sensitivity and 100% specificity. But it was a moderate marker to differentiate between lower and upper grades of OSMF though with an excellent sensitivity of 100% but with a poor specificity of 42.1% showing an average positive predictive value of 62% but an absolute negative predictive value limiting its role as a screening tool only. The other part of the study showed that FDP expression was an excellent marker to differentiate between control and OSMF patients with a sensitivity of 94.6%, specificity of 100%, positive predictive value of 100% and negative predictive value of 94.9%. FDP expression was a mild marker to differentiate between early and advanced stages of OSMF with a high sensitivity and a low specificity thus limiting its role as a screening tool only with a high incidence of false positivity.

FDP quantification, on the other hand was found to be a good tool to differentiate between early and advanced stages of OSMF with a sensitivity of 88.9%, specificity of 63.2%, positive predictive value of 69.6% and negative predictive value of 85.7%. Of the two tests, the diagnostic accuracy of FDP levels in relation to salivary coagulopathy procedure was 91.9% which offers better prospects of FDP levels over salivary coagulopathy estimation procedures. The estimation of FDP levels may be taken as an indicator of the gravity of this disease condition as the levels of FDP increased with increase in the clinical grading of OSMF. However, further research must be directed in assessing the exact nature of this unusual feature of salivary coagulopathy among OSMF patients and its role in causation of OSMF.

## Conclusion:

There is a positive correlation between the severity of the disease and the formation of salivary coagulum in patients with oral submucous fibrosis. A significant inter-stage difference is seen for FDP levels in OSMF patients. It is seen to be higher in higher stage and lower in lower stage. Of the two tests, FDP quantitation is a more accurate test to clinically assess OSMF than salivary coagulopathy assessment.

## Declarations:

## Trial registration:

Not applicable.

## Ethics approval:

The ethical approval was taken from Institutional Ethics Committee of Kothiwal Dental College and Research Centre, Moradabad, Uttar Pradesh, India.

## Consent to participate:

The written informed content was taken from all participants of the study.

## Data availability:

All data has been produced in the manuscript. The raw files are with the corresponding author and can be produced upon request.

## Consent for publication:

The Institutional Ethics Committee of Kothiwal Dental College and Research Centre, Moradabad, Uttar Pradesh, India gave the consent to publish the findings. No information pertaining to patient identification has been revealed anywhere in the manuscript.

## Funding:

None are declared.

## Figures and Tables

**Table 1:** Comparison of salivary coagulopathy in different clinical stages of OSMF

**SN**	**Condition**	**Stage I (n=8)**		**Stage II (n=11)**		**Stage III (n=10)**		**Stage IV (n=8)**		**Significance of difference**	
		**No.**	**%**	**No.**	**%**	**No.**	**%**	**No.**	**%**	**χ^2^**	**P**
1	t_1_	4	50	7	63.6	10	100	8	100	10.178	0.017
2	t_2_	0	0	0	0	0	0	0	0	0	1
3	t_3_	0	0	0	0	0	0	0	0	0	1
4	t_4_	0	0	4	36.4	7	70	7	85.5	14.902	0.002

**Table 2:** Correlation of FDP expression in relation to salivary coagulopathy

***FDP Expression***	**Coagulopathy**		**Total**
	**Positive**	**Negative**	
Positive	29	6	35
Negative	0	39	39
Total	29	45	74

## References

[1] Shah N, Sharma PP (1998). J Oral Pathol Med..

[2] Pindborg JJ (1984). Scand J Dent Res..

[3] Ho PS (2007). Oral Surg Oral Med Oral Pathol Oral Radiol Endod..

[4] Haque MF (2000). J Oral Pathol Med..

[5] Chatuvedi VN (1991). J Indian Dent Assoc..

[6] Kadani M (2014). J Clin Diagn Res..

[7] Ghom AG (2007). Textbook of Oral Medicine..

[8] Pinakapani R (2009). J Ind Acad Oral Med Rad..

[9] Gupta S (2014). J Clin Diagn Res..

[10] Tupkari JV (2007). JIAOMR..

[11] Rajalalitha P, Vali S (2005). J Oral Pathol Med..

[12] Pindborg JJ, Sirsat SM (1966). Oral Surg Oral Med Oral Pathol..

[13] Dave RP (1987). J Ind Dent Assoc..

[14] Anuradha CD, Devi CS (1993). Indian J Med Res..

[15] Mathur RM, Jha T (1993). J Ind Dent Asso..

[16] Haider SM (2000). Br J Oral Maxillofac Surg..

[17] Gupta S (2004). Indian J Clin Biochem..

[18] Ahmad MS (2006). J Indian Soc Pedod Prev Dent..

[19] Kumar A (2007). Oral Surg Oral Med Oral Pathol Oral Radiol Endod..

[20] Kiran Kumar K (2007). Indian J Dent Res..

[21] Zhang X (2008). J Oral Pathol Med..

[22] Chaturvedi VN, Marathe NG (1988). The Indian Practitioner..

[23] Khanna JN, Andrade NN (1995). Int J Oral Maxillofac Surg..

[24] Lai DR (1995). J Oral Pathol Med..

[25] RAO AB (1962). Br J Surg..

[26] Pindborg JJ (1968). Br J Cancer..

[27] Canniff JP (1986). Br Dent J..

[28] Pathak AG (1984). Ind J Otolaryngol..

